# Behavioral Training Related Neurotransmitter Receptor Expression Dynamics in the Nidopallium Caudolaterale and the Hippocampal Formation of Pigeons

**DOI:** 10.3389/fphys.2022.883029

**Published:** 2022-05-04

**Authors:** Christina Herold, Philipp N. Ockermann, Katrin Amunts

**Affiliations:** ^1^ C. & O. Vogt-Institute for Brain Research, Medical Faculty, University Hospital and Heinrich-Heine University Düsseldorf, Düsseldorf, Germany; ^2^ Institute of Neuroscience and Medicine INM-1, Research Center Jülich, Jülich, Germany

**Keywords:** hippocampus, prefrontal cortex, stimulus-response learning, reward learning, decision-making, associative learning, avian, quantitative real-time PCR

## Abstract

Learning and memory are linked to dynamic changes at the level of synapses in brain areas that are involved in cognitive tasks. For example, changes in neurotransmitter receptors are prerequisite for tuning signals along local circuits and long-range networks. However, it is still unclear how a series of learning events promotes plasticity within the system of neurotransmitter receptors and their subunits to shape information processing at the neuronal level. Therefore, we investigated the expression of different glutamatergic NMDA (*GRIN*) and AMPA (*GRIA*) receptor subunits, the GABAergic *GABARG2* subunit, dopaminergic *DRD1*, serotonergic *5HTR1A* and noradrenergic *ADRA1A* receptors in the pigeon’s brain. We studied the nidopallium caudolaterale, the avian analogue of the prefrontal cortex, and the hippocampal formation, after training the birds in a rewarded stimulus-response association (SR) task and in a simultaneous-matching-to-sample (SMTS) task. The results show that receptor expression changed differentially after behavioral training compared to an untrained control group. In the nidopallium caudolaterale, *GRIN2B*, *GRIA3*, *GRIA4*, *DRD1D*, and *ADRA1A* receptor expression was altered after SR training and remained constantly decreased after the SMTS training protocol, while *GRIA2* and *DRD1A* decreased only under the SR condition. In the hippocampal formation, *GRIN2B* decreased and *GABARG2* receptor expression increased after SR training. After SMTS sessions, *GRIN2B* remained decreased, *GABARG2* remained increased if compared to the control group. None of the investigated receptors differed directly between both conditions, although differentially altered. The changes in both regions mostly occur in favor of the stimulus response task. Thus, the present data provide evidence that neurotransmitter receptor expression dynamics play a role in the avian prefrontal cortex and the hippocampal formation for behavioral training and is uniquely, regionally and functionally associated to cognitive processes including learning and memory.

## Introduction

Learning and cognitive processes in mammals result in changes the way neurons communicate to support memory and/or other cognitive functions (Snowball et al., 2013; Kelly et al., 2014; Vijayvagharan et al., 2017; Cools and Arnsten, 2021; Faraza et al., 2021). Different mechanisms like long-term potentiation (LTP) and long-term depression (LTD) play a pivotal role in synaptic plasticity to strengthen or lower synaptic contacts of neurons. These processes are strongly linked to changes of the neurotransmitter receptor composition at the synapse, while receptors are differentially expressed in different brain areas and networks (Kandel et al., 2021 [1981]; Lein et al., 2007; Zilles et al., 2015; Zilles and Amunts, 2009; Zilles and Palomero-Gallagher, 2017; Palomero-Gallagher et al., 2020).

The prefrontal cortex and the hippocampus are two key brain areas involved in learning and cognitive processes. While the prefrontal cortex supports functions like working memory, response selection, decision making and reward-guided learning, reward evaluation and prediction (Miller and Cohen, 2001; Fuster, 2015 [1980]; Roberts et al., 1998; Friedman and Robbins, 2021), the hippocampus is predominantly involved in episodic memory, memory consolidation, spatial memory and navigation, pattern separation, context-dependent and associative learning (Brasted et al., 2003; Broadbent et al., 2004; Eichenbaum, 2004; Suzuki, 2005; Andersen et al., 2007; Bird and Burgess, 2008; Tyng et al., 2017). In mammals, it has been shown that different cognitive functions and the performance in cognitive tasks are associated with changes in neurotransmitter receptor binding (Zilles et al., 2000; Takahashi et al., 2008; McNab et al., 2009; Paoletti et al., 2013; Wass et al., 2018). Those studies have demonstrated in mammals that learning and consecutive exercise of cognitive tasks can result in an increase or a decrease of neurotransmitter receptors that was correlated to different behavioral tests, while less information was provided about altered subunit composition or even subtypes, which may change the signal propagation at the synapse. Further, it is yet still not fully understood how different learning processes and cognitive tasks are accompanied by receptor expression dynamics to support a certain behavioral change *via* learning processes and how this is associated to certain brain areas like the prefrontal cortex and the hippocampus.

In the avian brain, the nidopallium caudolaterale (NCL) is a brain structure at the caudal end of the forebrain that shares many cellular, anatomical, neurochemical, electrophysiological and functional similarities with the mammalian prefrontal cortex, although the evolution of the pallial organization and neuronal development in birds was separated from that of mammals during the past 310 million years (Waldmann and Güntürkün, 1993; Kumar and Hedges, 1998; Puelles et al., 2000; Güntürkün and Bugnyar, 2016; Medina et al., 2019; Stacho et al., 2020). Like the mammalian prefrontal cortex, the avian NCL is a highly interconnected associative forebrain area. It receives projections from all sensory modalities (Kröner and Güntürkün, 1999), projects to premotor areas (Kröner and Güntürkün, 1999), is one of the main targets of dopaminergic innervation (von Eugen et al., 2020) and shows similarities to the neurotransmitter receptor profile of prefrontal cortical areas of mammals (Herold et al., 2011). Similar to the prefrontal cortex, lesions of the NCL resulted in deficits in delayed alternation tasks, reversal learning and response selection (Mogensen and Divac, 1982; Hartmann and Güntürkün, 1998; Diekamp et al., 2002).

Electrophysiological recordings in the NCL proved working memory neurons firing during delay (Diekamp et al., 2002; Veit et al., 2014), which is accompanied by an increase of dopamine (Karakuyu et al., 2007), as well as firing of more general “executive control” neurons that encode what to forget and what to remember (Rose and Colombo, 2005). Further, reward-related neurons (Kalenscher et al., 2005; Koenen et al., 2013; Starosta et al., 2013; Dykes et al., 2018) and neurons that encode the association of new stimuli (Veit et al., 2015), numbers, categories or abstract rules were detected in the NCL (Kirsch et al., 2009; Veit and Nieder, 2013; Ditz and Nieder, 2015; 2016). Additionally, blockade of dopamine D_1_-receptors in the NCL has been shown to decrease performance in reversal learning and working memory (Diekamp et al., 2000; Herold et al., 2008), and infusion of glutamatergic NMDA receptor antagonists showed a role for NMDA receptors in response selection and cognitive flexibility (Lissek and Güntürkün, 2004; Herold, 2010). It has also been shown that dopamine D_1_-receptors were differentially expressed in the NCL and the striatum after behavioral training in a set of learning paradigms that distinguished stimulus-response association, response selection and maintenance of stimuli information like color, i.e., working memory (Herold et al., 2012). Based on these many resemblances between the mammalian prefrontal cortex and the NCL, the NCL is considered to be the avian prefrontal cortex (Güntürkün, 2005; Güntürkün and Bugnyar, 2016).

The avian hippocampal formation (HF) develops from the medial pallium and shares the origin with the mammalian hippocampus (Medina and Abbelan, 2009; Puelles, 2011). However, the avian hippocampal formation and the mammalian hippocampus show a different morphology (Atoji and Wild, 2004; Herold et al., 2014; Striedter, 2016). It is still under debate which subdivision of the avian hippocampal formation corresponds to its mammalian counterpart or even exists (Aboitiz et al., 2003; Colombo, 2003; Kempermann, 2012; Tosches et al., 2018; Bingman and Muzio, 2017; Barnea and Smulders, 2017; Herold et al., 2015; 2019). Apart from these controversial views, of what is known so far, cell types, connectivity and functionality of the hippocampal formation in birds largely matches the mammalian situation (Wieraszko and Ball, 1991; Colombo and Broadbent, 2000; Macphail, 2002; Bingman et al., 2005; Atoji and Wild, 2006; Shanahan et al., 2013; Herold et al., 2015, 2019; Gagliardo et al., 2020). Similar to mammals, the hippocampal formation of birds is involved in spatial cognitive processes and episodic memory and orientation that includes learning new routes/maps or food caches as well as recall familiar locations or behavioral inhibition (Sherry et al., 1992; Hampton and Shettleworth, 1996; Clayton and Dickinson, 1998; Mayer et al., 2010; Scarf et al., 2014; Gagliardo et al., 2020). These functions are further accompanied by adaptions in volume and adult neurogenesis (Smulders et al., 1995; Barnea and Pravosudov, 2011; Barkan et al., 2017). Recordings of neurons in the hippocampal formation of birds revealed visually responsive cells (Scarf et al., 2016; Ditz et al., 2018), spatially responsive cells (Hough and Bingman, 2004; Sherry et al., 2017), place cells (Payne et al., 2021) and head direction cells (Ben-Yishay et al., 2021). Properties of avian hippocampal cells seem to be very similar to mammalian hippocampal cells, which is also reflected in the neurotransmitter receptor fingerprints of hippocampal regions across species (Kahn et al., 2008; Herold et al., 2014; 2015). In addition, pharmacological approaches have shown that the blockade of noradrenergic receptors results in deficits of memory formation, while NMDA-receptor blockade seems to be involved in memory consolidation but not formation (Gibbs et al., 2008). This is in line with the finding that LTP occurs in the hippocampus of birds, but seems to be independent of NMDA-receptor activation. This in turn implies a possible other form of synaptic plasticity in the hippocampus of birds (Margrie et al., 1998).

Taken together, both the avian prefrontal cortex and the hippocampal formation seem to be relevant for learning and memory, while the functional resemblance between prefrontal and hippocampal areas of mammals and birds and the underlying mechanisms are still poorly understood.

Here we aim to disentangle the role of receptor plasticity in the NCL and the HF after learning a rewarded response-association task and a stimulus-selection and -comparison task. The latter task includes the components of the first task but adds the component of stimulus comparison and selection, i.e., decision-making. Therefore, we measured the expression levels of different glutamatergic NMDA (*GRIN*) and AMPA (*GRIA*) receptor subunits, GABAergic *GABARG2* subunit, dopaminergic *DRD1A* (*DRD1*), DRD1B (*DRD5*), *DRD1D* serotonergic *5HTR1A* and noradrenergic *ADRA1A* in both areas with quantitative real-time PCR after training the birds in these two tasks. Apart from the above-mentioned involvement of these receptor types in different behavioral tasks tested with pharmacologically manipulated animals, we choose them for our investigation out of three different reasons:

(1) The ionotropic glutamatergic NMDA and AMPA receptors are known key receptors involved in LTP and LTD during learning (Bliss and Cooke, 2011). The function of NMDA glutamate receptors changes according to their sub composition which is rather complex as seven subunits with different isoforms exist. In a nutshell, NMDA receptors form heteromeric complexes (tetramers) with different subunits that can change the affinity for ligands and channel properties (Seeburg, 1993). Particularly the GRIN1/GRIN2B composition has been implicated in learning and memory of mammals and GRIN2B mediates higher cognitive function in the prefrontal cortex (Wang et al., 2013). For example, higher amounts of the GRIN2B subunit result in higher opening probabilities of the ion-channel compared to GRIN2C and both exhibit lower opening probabilities compared to GRIN2A, which results in different steady-state activities or “burst” activities (Wyllie et al., 2013). Further, reduced levels of GRIN2C in the medial prefrontal cortex of mice lead to higher inhibition, reduced dendritic spine density and impairments in working memory, associative learning and sensorimotor gating (Gupta et al., 2016). GRIN3A is critical to from long-term synaptic plasticity for memory consolidation, while the function of GRIN3B is less clear and mostly confined to motor neurons, where they are highly expressed in contrast to low cortical expression sites in mammals (Das et al., 1998; Niemann et al., 2007; Roberts et al., 2009; Pachernegg et al., 2012). AMPA receptors also form heterotetramers comprised out of the four subunits GRIA1-4 (Hollmann and Heinemann, 1994). They mediate synaptic strengthening subunit specific due to LTP and LTD (Kessels and Malinow, 2009). For example, increased levels of GRIA2/GRIA3 can be found in cortical synapses of mice when deprived from experience-dependent stimulation (Makino and Malinow, 2011). In the hippocampus of mammals, GRIA1 has been further described to be important for LTP maintenance (Jiang et al., 2021) and GRIA1 expression is important for short-term memory, while depletion of GRIA1 results in profound long-term memory (Bannerman et al., 2014).
(2) Neurotransmitters like noradrenaline, dopamine and serotonin act via their specific receptors as modulators of synaptic transmission and play a key role for different aspects in learning and memory. Both, the NCL and the HF receive dopaminergic, noradrenergic and serotonergic input (Reiner et al., 1994; Challet et al., 1996; Durstewitz et al., 1999). Thereby, metabotropic ADRA1A regulate hippocampal interneurons by depolarizing and releasing GABA, adult neurogenesis and short-term plasticity, while increased levels of ADRA1A improved LTP in the mouse hippocampus and cognitive performance (Perez, 2020). All dopamine receptor D1 subtypes are metabotropic and involved in reward-related learning processes and goal-directed behavior, in both, prefrontal and hippocampal areas (Goto and Grace, 2005; Herold et al., 2012; Puig et al., 2014). Hippocampal metabotropic 5HTR1A play a role in mice adult neurogenesis (self-renewal of precursor cells; Gould, 1999; Klempin et al., 2010), spatial learning (Glikmann-Johnston et al., 2015) and can be modulated in birds by ingestive and sleep behavior (Hoeller et al., 2013; Dos Santos et al., 2015).
(3) Because we found that *DRD1A* (*DRD1*), *DRD1D* receptor expression was altered in the NCL by a similar training protocol (Herold et al., 2012), we wanted to know if further interacting neurotransmitter receptors that can shape and spatially tune the signal transfer along dendrites are additionally affected, i.e., GRIN1 interacts with DRD1 and GABARG2 interacts with DRD5 (Wang et al., 2008; Puig et al., 2014).

We hypothesized that if receptor plasticity plays a role in these components of learning and cognitive functions, we would see altered expression levels as compared to untrained controls. Moreover, if the NCL and the HF would play a differential role in these functions, we would expect that the expression levels change specifically according to the task. Additionally, we would be able to discover in detail which receptors or subunits are involved in these processes in the avian brain and compare it to mammals, to gain more insights into the fundamentals of learning.

## Materials and Methods

### Experimental Design

**FIGURE 1:**
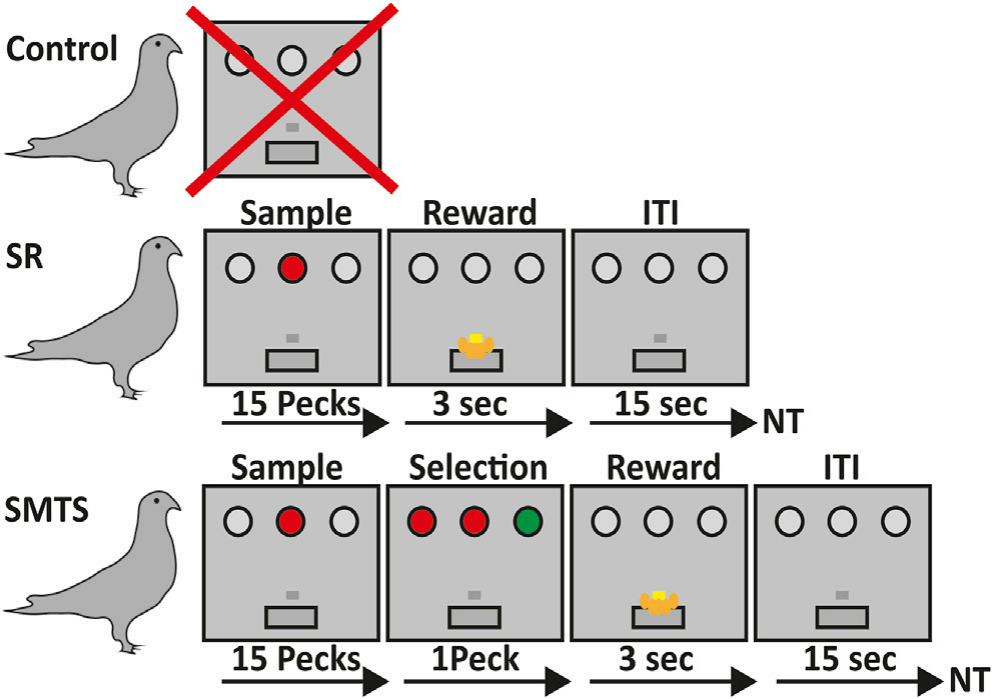
Overview of the learning paradigms for the three pigeon groups. Birds in the control group did not participate in any operant task (line 1). Pigeons of the stimulus-response (SR)-group were trained to associate a stimulus with a reward after 15 pecks on either a red or green operant key on one of the three positions at the back wall of the Skinner box, which was then rewarded for 3 s with access to food *via* a food hopper. After that an intertrial interval (ITI) of 15 s began before the next trial (NT) started (line 2). In the simultaneous-matching-to-sample (SMTS)-group pigeons were trained to peck 15 times on the either red or green illuminated center key. Afterwards a choice period started, during which they had to select the lateral key that matched the sample color. During this phase all keys were simultaneously illuminated so that the birds were able to compare the stimuli and decide. Selection of the correct response resulted into 3 s access to food that was followed by an ITI of 15 s before the NT started (line 3).

Thirty adult, age-matched (3–4 years), unsexed pigeons (*Columba livia*) of the local stock were used in the experiments. Each test-group included ten pigeons. At the beginning, none of the birds had participated in an experiment before. The pigeons of the control group did not undergo any behavioral training, while the remaining two groups, the stimulus-response group (SR) and the simultaneous-matching-to-sample (SMTS) group, performed task dependent training sessions (Figure 1). During the experiments, all pigeons were housed in individual cages in a temperature-controlled room on a 12-h light-dark cycle. One week before the experiment started, all birds were food-deprived to 80% of their normal free feeding weights but had *ad libitum* access to water and grit. Animals were trained four to 5 days a week in an operant chamber. After each training session animals were compensated for food to nullify the differences between food-intake in the operant chamber between animals. The duration of sessions was equal for all stages according to the training task protocol. After reaching the learning criterion of the SR or the SMTS pigeons were directly used for brain tissue preparation. In parallel, pigeons of the control group were housed in individual cages for 8 weeks under the same conditions like the pigeons of the SR and the SMTS group before they were sacrificed. All pigeons were handled, controlled for weight every day and fed.

### Ethics Statement

The animal procedures were conducted in accordance with the NIH Guide for the Care and Use of Laboratory Animals and under adherence to the German laws to protect animals, and hence, the European Communities Council Directive of 18 June 2007. The experimental protocol was approved by national authorities and the Ethics Committee of the Landesamt für Natur, Umwelt und Verbraucherschutz (LANUV) of North Rhine-Westphalia, Germany.

### Learning Environment

Two operant chambers (34 cm × 33 cm × 36 cm) were utilized for the learning procedures and behavioral training. Each chamber was controlled *via* a digital input-output board (CIO-PDISO8; Computer Boards, Inc. United States) and illuminated by a 24 W, centrally fixed light bulb. Three opaque operant keys (2 cm in diameter) with a distance of 10 cm in-between were located at the back panel of each box, 22 cm above the floor. The pecking keys were homogeneously illuminated either by white, red or green light, without matching the brightness of colors. White lights were used in the operant conditioning and pre-training sessions, while red and green lights were used during the test sessions in SR and SMTS tasks. The food-hopper, which was combined with a light-emitting diode, was fixed in the center of the back panel, 5 cm above the floor.

### Behavioral Tasks

The rational and the sequential procedure of the learning tasks is depicted in Figure 1.

#### Pre-Training

During the first sessions pigeons received auto-shaping. After they started pecking the key during auto-shaping sessions, they were trained to peck reliably on the center key, whenever it was illuminated with white light. After a single peck, the light of the center key turned off, and the pecking was reinforced with three seconds access to food, followed by an inter-trial interval of 15 s. In the next steps, each trial began with the illumination of the center key. One peck on the lateral keys during this phase terminated the trial that was then followed by an inter-trial interval of 15 s and a retry of the trial. One training session included 80 trials with a 15 s inter-trial interval between each trial. In the following training sessions, the number of pecks required on the center key to extinguish the center light was constantly increased from 1 to 15 pecks. The criterion for the pre-training was 100% correct responses in one session. After this training, pigeons were randomly divided into two groups. One group was trained further in a colored stimulus-response task and the other group had to learn a simultaneous-matching-to-sample task.

#### The Colored Stimulus-Response Task

After pre-training, pigeons were trained for a stimulus-response (SR) task with colored operant keys. For this step, they learned to peck reliably on one of the three keys (two lateral, on central), whenever it was illuminated with colored light. No discrimination or response selection of colors was involved. After 15 pecks, the light was turned off, and birds were rewarded with 3 s’ access to food, followed by an inter-trial interval of 5 s. Illumination of the three keys was randomized to exclude a spatial bias for one of the keys. Pecking one of the dark keys caused punishment by a 10 s’ time-out period during which all lights in the operant chamber were turned off. One session included 80 trials with a 15 s inter-trial interval between each trial. Before decapitating the animals for quantification of the different neurotransmitter receptor mRNA levels, all pigeons had to reach an overall criterion of 80% correct responses on three subsequent training days (SR-group). Here, the SR task included the association of a colored stimulus with a response, i.e., track a location of the colored key, repeat pecking to it 15 times to then obtain a reward, which further includes processes like certain reward expectation and reward consumption because a classical operant conditioning procedure was applied here (Figure 1).

#### SMTS Task

After pre-training, the operant keys were illuminated with colored light and pigeons were trained to peck on the colored key like the SR-group. After reaching the criterion, the illumination of the central stimulus with either red or green light started each trial. The center light stayed on until the pigeon had pecked the key 15 times. Immediately thereafter, the two lateral choice keys were illuminated simultaneously, one in red and the other in green light, while the central key stayed on. Pigeons were rewarded after pecking the lateral illuminated choice key that matched the color of the simultaneously illuminated central key with 3 s’ access to food, and were punished after pecking the non-matching key with a 10 s’ time-out in darkness. No working memory of stimulus color information was required to perform the task because during the choice phase all keys stayed on. One session included 80 trials with a 15 s inter-trial interval between each trial. Training was conducted until the pigeons reached a performance level of 80% correct responses on three subsequent days. The colors of the keys were randomly presented to avoid that the pigeons learn a fixed sequence of presentation of the stimuli. Here, the SMTS task required the same motor skills like the SR task until the 15th peck on the central key. The learning component that came on top, in comparison to the SR-group, was that immediately after they pecked the central key 15 times, pigeons had to match the color of the central key to one of the lateral choice keys and then select and initiate a response to the identified key before they had the chance to obtain the reward (Figure 1). Thus, relative to the SR birds, the SMTS group had to learn a color dimension, discriminate and match between the two colors, which further included a response selection and decision-making task component. In contrast to the SR task, in the SMTS task the reward was not guaranteed but choice dependent.

### RNA Preparation and Quantitative Real-Time RT-PCR

**FIGURE 2:**
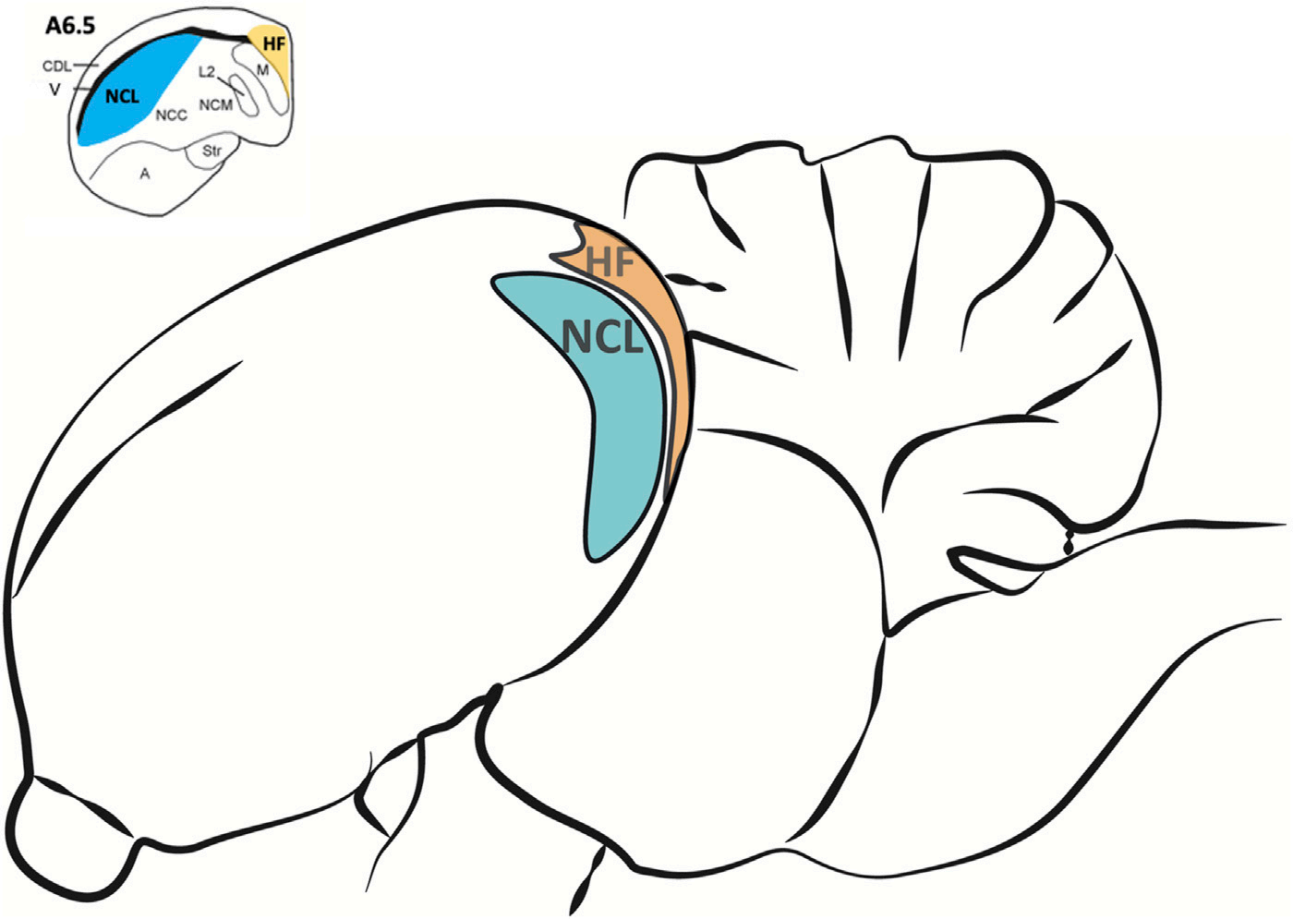
Regions of interest for the analysis of different neurotransmitter receptor mRNA levels in the pigeon brain after learning. The nidopallium caudolaterale (NCL, blue) of pigeons is a half-moon shaped structure located laterally beneath the forebrain ventricle at the end of the caudal forebrain (see upper left). It is involved in executive functions and reward processing, comparable to the prefrontal cortex in mammals. The hippocampal formation (HF, orange) is located above the forebrain ventricle and begins at the most caudal level end of the avian telencephalon along the medial wall up to the anterior level A 9.00 (Herold et al., 2014). Here we only used HF samples caudal from A 6.50 (see upper left) because the largest portions of both, NCL and HF, are located beyond.

For brain tissue preparation pigeons were deeply anesthetized with Equithesin (0.5 ml/100 g body weight, i.m.) and decapitated. Brains were quickly removed and stored on ice. The NCL and the HF (Figure 2) were dissected out, frozen in liquid nitrogen and stored at −80°C for later use. For the dissection, the pigeon brain was adjusted under a binocular microscope with a µm scale and cut into 2 mm thick slices. The NCL was prepared according to Herold et al. (2011). Because a large part of the half-moon-shaped NCL is located caudal from the stereotactic coordinate A 6.25, we used the slices caudal from this coordinate and removed the ventrally positioned arcopallium/amygdala complex (Herold et al., 2018). The tractus dorso-arcopallialis served for orientation because it is well visible in the native preparation. Next, we cut off the medial parts of the nidopallium, i.e., the nidopallium caudolaterale central and the nidopallium caudomediale as well as the overlaying dorsolateral corticoid area region that is, like the HF, separated from the NCL by the ventricle. Therefore, this sample consists mostly of NCL material. The HF was prepared according to Herold et al. (2014) as the most medial portion above the ventricle from atlas coordinates A 6.25 to A 3.00 along the anterior-posterior axis and M 0.00 to M 2.00 along the medial-lateral extension in coronal slices (Karten, 1967). Total RNA of all probes was extracted to process for real-time RT-PCR by using the NucleoSpin®RNA II Kit (Macherey-Nagel, Düren, Germany). RNA quality was checked for each probe photometrically. Because of technical issues, one HF-probe of the SR group and two HF-probes of the SMTS group were lost (not enough RNA could be eluted). For the rest of the probes, cDNA was obtained with the Superscript™II RT First Strand Synthesis System for RT-PCR (Invitrogen, Karlsruhe, Germany). For each probe 600 ng of total RNA was used for the RT reaction. Each probe was replicated twice.

Real-time PCR was performed on a LightCycler^®^ 480 system (Roche, Mannheim, Germany) to determine the mRNA expression in the NCL or HF. For the preparation of the PCR standard reaction the protocol from LightCycler^®^ FastStart DNA Master^PLUS^ SYBR Green I (Roche, Mannheim, Germany) at a total volume of 20 µl was used. For each sample 1 µl cDNA diluted with 4 µl PCR-grade water was used as template for the reaction, with 10 µm forward and backward primers. Both targets and reference amplifications were performed in doublets in separate wells on a 96 well plate. The primers for the different neurotransmitter receptor subtypes and the “housekeeping genes” H3 histone3B (*H3-3B*) and Glyceralaldehyde-3-Phosphate dehydrogenase (*GAPDH*) used in the real-time PCR are listed in Table 1. The thermal cycling conditions included 10 min at 95°C pre-incubation, followed by 40 amplification cycles comprising 95°C for 10 s, 62°C for 10 s, and 72°C for 20 s, and one cycle for melting curve analysis comprising 95°C for 0 s, 65°C for 15 s, and 95°C with a slope of 0.1°C/s, followed by cool-down to at least 40°C. Under these conditions the efficiency for all primers was in the range of two, i.e., at maximum. In addition, the expression of the reference genes was controlled in all groups. None of the groups showed regulation in *H3-3B* or *GAPDH* expression, and all expression data of the receptor subtypes were normalized to both of these reference genes.

**TABLE 1:** Primerpairs used for quantitative RT-PCR. Each primer pair binds specifically the indicated gene without cross-reactions. The obtained fragments were verified by sequence analysis.

Gene	Forward primer 5′-3′	Reverse primer 5′-3′	GenBank accession # for amplicon	Size (bp)
*DRD1(D1A)*	ATA​CGC​CGC​ATC​TCA​GCC​TT	TCT​GTT​GCC​GGT​CGT​GTT​CT	XM_021289222.1	72
*DRD5(D1B)*	TAG​TCA​TGC​CCT​GGA​AGG​CG	ATG​GAG​GCC​GTG​GAA​CAC​AT	XM_021285849.1	106
*DRD1D*	AGC​CCC​AAG​AGC​CAT​CAG​AC	GGG​TGA​TCG​GGT​TCC​ACA​CA	XM_021288368.1	84
*GRIA1*	GCA​CTG​AGA​GGT​CCC​GTA​AA	TAG​AAA​ACC​CCG​GCC​ACA​TT	NM_001282812.1	170
*GRIA2*	TAC​GGC​ATC​GCC​ACA​CCT​AAA	GGG​CGC​TGG​TCT​TCT​CCT​TAC	NM_001315518.1	165
*GRIA3*	AAG​GGC​AAG​TTC​GCC​TTC​CT	CTT​GGA​ATC​CAG​GTT​GCC​GC	XM_021290293.1	102
*GRIA4*	CGT​GTC​CGC​AAA​TCC​AAG​GG	TCC​TTG​GAG​TCA​CCT​CCC​CC	XM_021286317.1	272
*GRIN1*	GGA​GGA​AGA​TGC​CCT​GAC​CC	CCT​TCT​CCG​ATG​CCG​GAG​TT	XM_013370181.2	78
*GRIN2B*	GCC​ATG​GCC​CTC​AGT​CTC​AT	GCC​ATG​TTC​TTG​GCT​GTC​CG	XM_013367952.1	245
*GRIN2C*	CGT​CAT​ACC​GGG​AGG​CTT​GT	CAG​GTA​GAG​GGG​CAG​GTT​GG	XM_021288660.1	98
*GRIN3A*	GCT​TTG​CCG​TCA​CAG​AGA​CC	ATT​CGT​GGT​AGG​ACC​AGC​CG	XM_021281606.1	139
*GRIN3B*	CGA​CTC​CGA​CTG​CAA​ACT​GC	AGA​TGC​CCA​TCT​GCA​GGG​TC	XM_021296971.1	221
*GABAAG2*	GCT​GCC​TGA​GCT​GAC​GTT​TC	AGA​ATG​CAG​TGC​TCC​CCA​GG	XM_005503517.2	273
*5HTR1A*	AAG​CGG​AGG​ATG​GCT​CTG​TC	GCC​ACT​TGG​GCA​TGT​AGC​AC	XM_021284843.1	148
*ADRA1A*	CCA​TCG​GGC​CTC​TTT​TTG​GC	GGG​CTC​CTC​GGT​GAT​CTG​AC	XM_021280945.1	74
*GAPDH*	CCA​TGC​CAT​CAC​AGC​CAC​AC	GGC​TGG​TTT​TTC​CAG​ACG​GC	NM_001282835.1	226
*H3.3B*	GTG​CAG​CCA​TCG​GTG​CGC​T	TGC​GAG​CCA​ACT​GGA​TAT​CT	EU196043.1	128

Real-time PCR products were verified by melting curve analysis, 2% agarose gel electrophoresis (ethidium bromide staining), and sequence analysis on an ABI PRISM Genetic Analyzer 3100C (Applied Biosystems, Darmstadt, Germany). All products matched the expected base pair length and sequence and showed no cross reaction.

### Data Analysis

For quantitative analysis of real-time RT-PCR data, the levels of target gene expression were normalized to the levels of the housekeeping genes histone *H3-3B* and *GAPDH* according to the following formula 2^−ΔCT^ with ΔCT = (Average of target gene CT_T_− average of housekeeping genes CT_HK_ for each probe). The resulting values were multiplied with 100 to achieve the percentage expressed values of each target gene from the two housekeeping genes. These values for each receptor subtype or subunit and each region were than statistically compared between groups with a non-parametric analysis with group as an independent factor (Kruskal-Wallis-ANOVA) because data was not normally distributed (Shapiro-Wilks’s test). When a group effect with Kruskal-Wallis was confirmed, a post-hoc analysis with Mann-Whitney- *U*-tests with continuity correction was conducted for pair-wise group comparisons resulting in corrected *p*- and z-values using Statistica 13.1 (StatSoft Europe GmbH, Hamburg, Germany). For the initial Kruskal-Wallis analysis between groups the *p*-level was set at 0.05. The post-hoc analysis results were additionally controlled with the Benjamini-Hochberg/Simes procedure for multiple comparisons for each individual gene and three groups that were tested. The analysis revealed that the acceptance *p*-level for a significant result of post-hoc analysis for the first comparison was *p* < 0.0166, for the second *p* < 0.033 and for the third *p* < 0.05.

## Results

**FIGURE 3:**
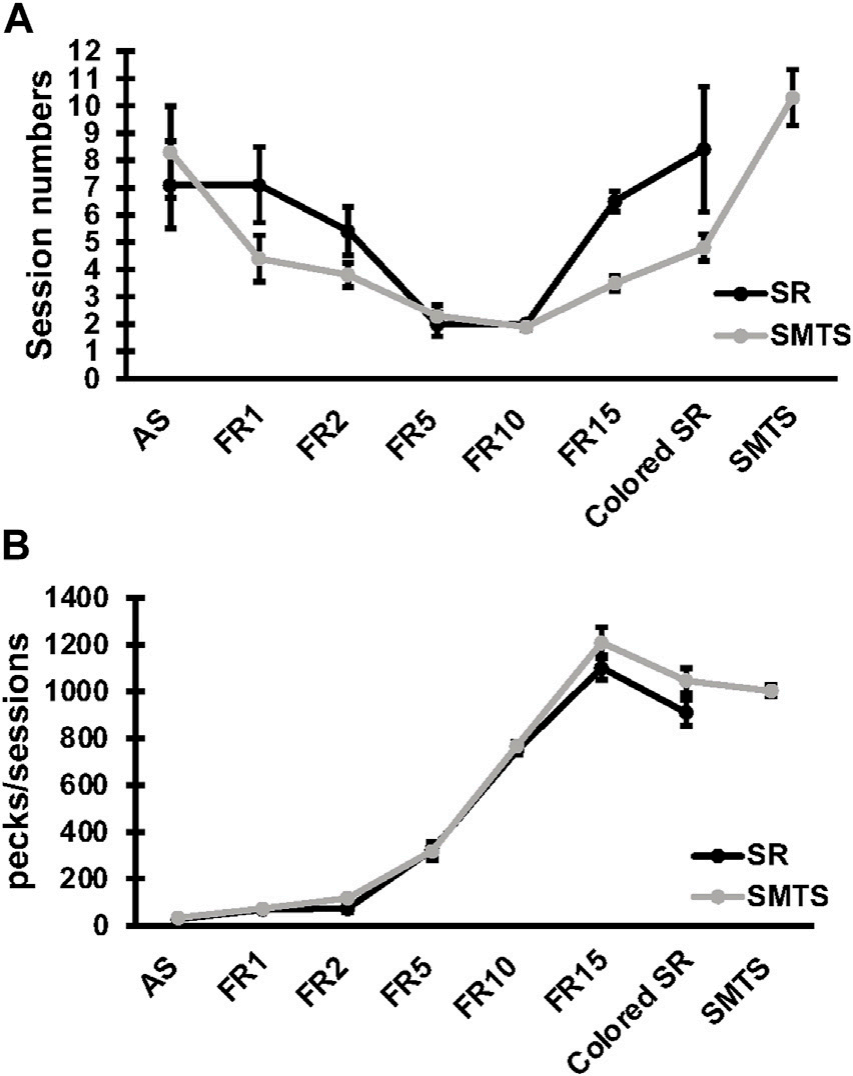
Learning curve and pecking activity of the two pigeon groups. The learning curve in **(A)** shows the session numbers for each step of the behavioral training by means ± SEM for each group (N = 10, SR and SMTS). At the end of the training all pigeons reached a performance level of 80% correct responses in both tasks (SR and SMTS). Both groups performed an equal total amount of sessions. In parallel, in **(B)** the pecking activity during the learning periods is presented by means ± SEM for each group (N = 10, SR and SMTS). Abbreviations: AS, autoshaping; FR1 to FR15, fixed ratio 1 to 15; Colored SR, Stimulus-Response task including color; SMTS, Simultaneous-Matching-To-Sample task.

Specific changes in expression for receptor subtypes and subunits in the NCL and the HF were observed in both experimental groups after a learning period of 9 ± 3 weeks (mean ± SD) of training (SR-group: 39 ± 13 total number of sessions in all learning tasks; SMTS-group: 40 ± 6 total number of sessions in all learning tasks, Figure 3A) when compared to control pigeons, which were untrained but had the chance to randomly peck grit and move in their cages. In addition, the number of pecks per session in each task was elucidated (Figure 3B). The mean number (over all learning tasks) of pecks per session was 3,252 ± 103 in the SR-group and 4,568 ± 136 in the SMTS-group, which additionally had to perform in the SMTS-task compared to the SR-group und thus carried out more pecks (Figure 3B). Until this point no differences in pecking activity could be observed (Figure 3B).

In the NCL the glutamatergic, dopaminergic and noradrenergic receptors showed significant changes, while in the HF the glutamatergic and the GABAergic receptors were altered. All expression data is presented as relative expression (%) of the two housekeeping genes *H3-3B* and *GAPDH*.

### Neurotransmitter Receptor Plasticity in the NCL After Associative Learning and Stimulus-Selection/Discrimination

The individual analysis with Kruskal-Wallis tests between groups for glutamatergic NMDA- and AMPA-receptor expression of the NCL showed significant differences for the *GRIN2B* subunit (H = 16.42, df = 2, N = 30, *p* = 0.0009), *GRIA2* (H = 6.89, df = 2, N = 30, *p* = 0.0319), *GRIA3* (H = 15.25, df = 2, N = 30, *p* = 0.0005) and *GRIA4* (H = 8.19, df = 2, N = 30, *p* = 0.0166), while the other glutamatergic receptors showed no effects between groups (*GRIN1*, *GRIN2C*, *GRIN3A*, *GRIN 3B*, *GRIA1*; *p* > 0.05).

**FIGURE 4:**
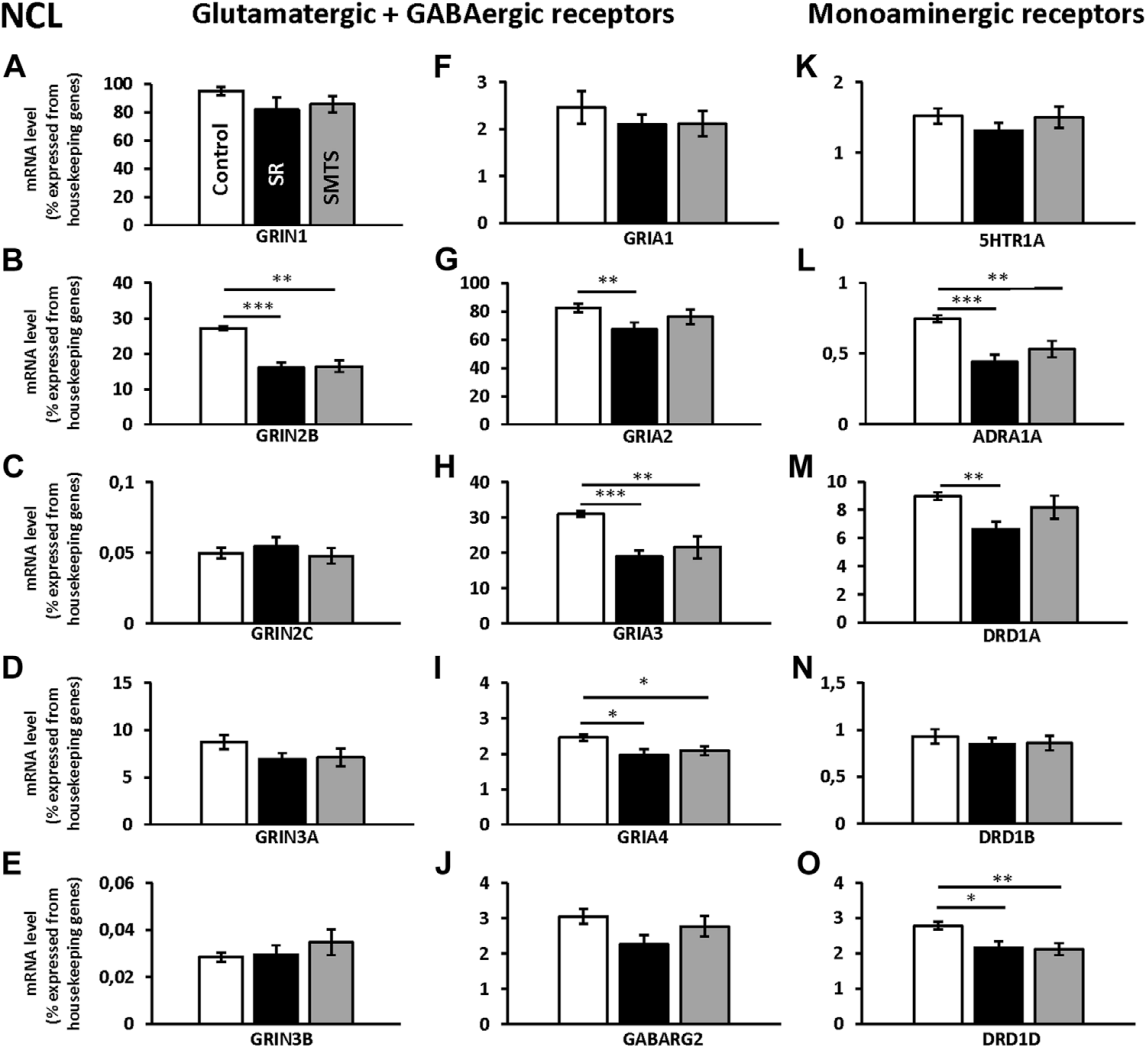
Neurotransmitter receptor expression in the nidopallium caudolaterale (NCL) after learning different tasks. The mRNA levels of 15 neurotransmitter receptors and subunits were quantified as relative expression levels in percent expressed of the two housekeeping genes *H3-3B* and *GAPDH*. Results for glutamatergic and GABAergic receptor mRNA levels in the control group (Control, white), the stimulus-response group (SR, black) and the simultaneous-matching-to-sample group (SMTS, grey) are presented in **(A–J)** and monoaminergic receptor mRNA levels in **(K–O)** (mean ± SEM; N = 10 for each group). Significant differences between the different learning groups are highlighted with asterisks (∗*p* < 0.03; ∗∗*p* < 0.01; ∗∗∗*p* < 0.001).

Post-hoc analysis with Benjamini-Hochberg/Simes correction revealed that *GRIN2B* expression decreased in both experimental groups (SR: z = 3.74, *p* = 0.0002; SMTS: z = 3.14, *p* = 0.0017) as compared to the control condition (Figure 4B). *GRIA2* decreased in the SR-group (z = 2.68; *p* = 0.007), while no effect was observed after SMTS-training (Figure 4G). *GRIA3* (SR: z = 3.59, *p* = 0.0003; SMTS: z = 2.99, *p* = 0.0028) and *GRIA4* were decreased in both experimental groups (SR: z = 2.53, *p* = 0.0113; SMTS: z = 2.31, *p* = 0.0211; Figures 4H,I).

The monoaminergic receptor expression differed between the three pigeon groups for the *ADRA1A* (H = 13.22, df = 2, N = 30, *p* = 0.0013), the *DRD1A* (H = 8.83, df = 2, N = 30, *p* = 0.0121) and *DRD1D* (H = 9.45, df = 2, N = 30, *p* = 0.0089) mRNA levels as revealed by Kruskal-Wallis tests.

The post-hoc analysis with subsequent Benjamini-Hochberg/Simes correction showed that ADRA1A were less expressed in the SR-group (z = 3.36, *p* = 0.0008) and SMTS-group (z = 2.61, *p* = 0.0091; Figure 4L) than in the control group. *DRD1A* decreased in the SR-group (z = 2.98, *p* = 0.0028; Figure 4M), while *DRD1D* was down-regulated in the SR- and the SMTS-group (SR: z = 2.49, *p* = 0.0126; SMTS: z = 2.68, *p* = 0.0073; Figure 4O) More general, in control pigeons, the highest expression levels were observed for *GRIN1* and *GRIA2* (Figures 4A,G), while the *GABARG2* subunit, i.e., was about 32 times lower expressed compared to *GRIN1*, and 27 times lower compared to *GRIA2* in the NCL (Figure 4J). From the monoaminergic receptors *DRD1A* showed the highest expression levels in the control group, followed by *DRD1D* and *5HTR1A* (Figures 4K,M,O).

### Changes in Expression Rates of Receptors in the HF After Associative Learning and Stimulus-Selection/Discrimination.

The comparisons with Kruskal-Wallis tests for the receptor expression rates in the HF of the three experimental pigeon groups showed differences for *GRIN2B* (H = 9.79, df = 2, N = 27, *p* = 0.0075), *GRIN3A* (H = 6.68, df = 2, N = 27, *p* = 0.0354), *GRIA3* (H = 6.05, df = 2, N = 27, *p* = 0.0484), *GABARG2* (H = 8.26, df = 2, N = 27, *p* = 0.0161) and *5HTR1A* (H = 6.42, df = 2, N = 27, *p* = 0.0404).

**FIGURE 5:**
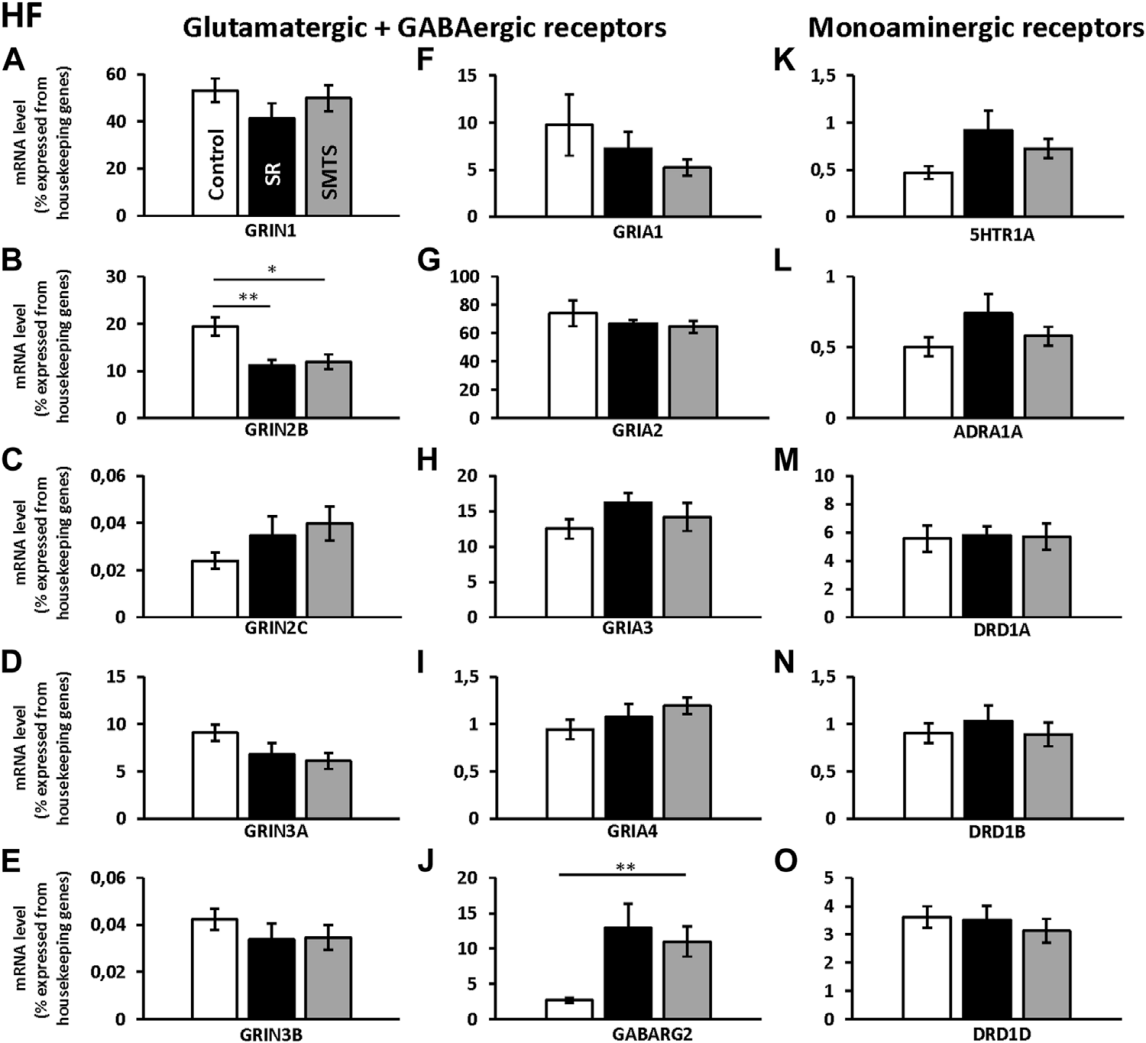
Neurotransmitter receptor expression in the Hippocampal formation (HF) after learning different tasks. The mRNA level of 15 neurotransmitter receptors and subunits were quantified as relative expression levels in percent expressed of the two housekeeping genes *H3-3B* and *GAPDH*. Results for glutamatergic and GABAergic receptor mRNA levels in the control group (Control, white), the stimulus-response group (SR, black) and the simultaneous-matching-to-sample group (SMTS, grey) are presented in **(A–J)** and monoaminergic receptor mRNA levels in **(K–O)** (mean ± SEM; Control: N = 10; SR: N = 9; SMTS: N = 8). Significant differences between the different learning groups are highlighted with asterisks (∗*p* < 0.03; ∗∗*p* < 0.01).

Post-hoc analysis and subsequent Benjamini-Hochberg/Simes correction showed that glutamatergic *GRIN2B* (SR: z = 2.74, *p* = 0.0062; SMTS: z = 2.44, *p* = 0.0145) were decreased under both training conditions compared to the control group, while *GRIN3A* levels have to be considered as not significant different (Control vs. SMTS: z = 2.36, *p* = 0.0185, n.s; Figures 5B,D). After correcting for multiple comparisons, *GRIA3* expression levels also showed no significant differences (Control vs. SR: z = −2.25, *p* = 0.0247, n.s; Figure 5H). GABAergic *GABARG2* subunit expression was increased in the SMTS-group (SMTS: z = −2.71, *p* = 0.0067; Figure 5J), while the comparison to the SR-group failed barely the significance criterion (SR: z =−2.08, *p* = 0.0373, n.s). The expression rates for *5HTR1A* receptors after SR-training had to be further considered equal after pair-wise comparison (SR: z = −2.25, *p* = 0.0247, n.s.; Figure 5K), while the other monoaminergic receptors also showed no effects (Figures 5L–O).

Regarding the glutamatergic receptors, the highest relative expression was detected for *GRIA2* (Figure 5G) followed by *GRIN1* (Figure 5A) and *GRIA3* (Figure 5H), while the lowest levels were measured for *GRIN2C* (Figure 5C) and *GRIN3B* (Figure 5E) in the HF of the control group. Overall, GABAergic *GABARG2* subunit expression was 28 times lower compared to *GRIA2* and 20 times lower compared to *GRIN1* expression. From the monoaminergic receptors, *5HTR1A*, *ADRA1A* and *DRD1B* showed the lowest expression rates in the HF of the control group (Figures 5K,L,N) and highest levels were seen for *DRD1A* and *DRD1D* (Figures 5M,O).

## Discussion

The study demonstrated receptor changes of different ionotropic and metabotropic neurotransmitter receptor subunits and subtypes in the NCL and HF of pigeons that were trained in two cognitive tasks: a rewarded color stimulus-response association task and a simultaneous matching to sample task that includes cognitive processes like stimulus-comparison and selection.

In the NCL, the NMDA-receptor subunit *GRIN2B*, the AMPA-receptor subunits *GRIA2*, *GRIA3* and *GRIA4*, the noradrenergic receptor subtype *ADRA1A* and the dopaminergic receptor subtypes *DRD1A* and *DRD1D* gene expression responded with a decrease after forming the rewarded stimulus-response association and behavioral training. The expression of these receptor was also decreased after SMTS training when compared to the control group, except for *GRIA2* and *DRD1A*, which showed no longer a difference to control levels after ongoing behavioral training to discriminate between two stimuli. In the HF, the mRNA levels of the NMDA-receptor subunit *GRIN2B* decreased after both, SR and SMTS training, while the GABA_A_-receptor subunit *GABARG2* increase was indicated after training of the operant stimulus-response task, but was statistically confirmed only after SMTS training.

To our knowledge, this is the first study that explored receptor expression dynamics of different neurotransmitter systems in the avian brain in dependence on learning and training of different tasks that constitute fundamentals in learning and cognition. The results underpin the importance of the avian prefrontal cortex and the HF to form stimulus-response associations that combines aspects of reward expectation and reward consumption with learning to respond to a certain stimulus on the one side, and to perform selective responses and stimulus-comparisons after the association has been established on the other. In both structures, receptor expression was initially altered after SR training. This “receptor state” seems to be mostly sustained and only slight adjustments have been found after SMTS training, while no differences between both learning paradigms were observed. However, specific receptor subtypes and subunits were altered to shape synaptic contacts according to the behavioral training and the expression levels changed differentially in the NCL and the HF. These results would argue against effects that could have been observed due to handling of the pigeons or higher pecking activity of the SR and SMTS group when compared to the control group. Further, pecks per sessions were equal between the two training groups until pigeons had to perform the SMTS-task. Nevertheless, we have to admit that SMTS- pigeons, although they did not perform in more sessions, executed more pecks in total compared to the SR-group and thus we cannot completely exclude that pecking (physical) activity influenced the expression of receptors in the SMTS-group. However, we would see other brain structures like the basal ganglia, the cerebellum or parts of the pre-motor arcopallium/amygdala complex predominantly involved in such motor behavior (Goodman et al., 1982; Yuan and Bottjer, 2020; Tian et al., 2022). Moreover, an earlier study of dopamine receptor expression after SR, SMTS and Delay-Matching-to-Sample training has shown that expression levels of receptors can change specifically according to different demands of cognitive tasks or learning components (Herold et al., 2012). Together, receptor expression changes can be associated with behavioral training and altered receptor expression may influence signal propagation at the neuronal level differentially in dependence of the structural involvement in a specific task.

### Receptor Plasticity in the Nidopallium Caudolaterale After Associative Learning and Stimulus Discrimination Training

The study showed that receptor plasticity in the NCL is linked to associative learning, which includes processes such as pairing the stimulus and the response, reward expectation and consumption and attention. In line with the present data, a role for the NCL in these processes has been confirmed in behavioral, pharmacological as well as electrophysiological experiments in different bird species (Koenen et al., 2013; Starosta et al., 2013; Veit et al., 2015; Kasties et al., 2016). Under the control condition, high levels of the NMDA receptor *GRIN1*, *GRIN2B*, AMPA receptor *GRIA2* and *GRIA3* subunits were detected, while the monoaminergic receptors were relatively low expressed, with the highest amounts found for *DRD1A* receptors. These findings are comparable to the receptor protein levels of NMDA and AMPA receptors, which were earlier investigated with non-selective ligands for specific subunits or subtypes (Rehkämper and Zilles, 1991; Herold et al., 2011). Additionally, low expression levels for the GABA_A_ receptor *GABARG2* subunit in the NCL were found, which corresponds to the general lower receptor protein concentrations of GABA_A_ receptors in the NCL compared to NMDA and AMPA receptors (Herold et al., 2011).

### Glutamatergic Receptors

After stimulus-response learning the receptor subunit/subtype expression profile of the NCL changed. From the nine investigated glutamatergic receptor subunits, four decreased (*GRIN2B, GRIA2, GRIA3, GRIA4*). Here, further evidence is provided that the decrease of different neurotransmitter receptors may be evoked by learning of the operant conditioning task and consecutive training to respond to a visual stimulus.

Considering that the changed glutamatergic neurotransmitter receptors are usually involved in increasing neuronal activity, it can be assumed that a decrease in transcripts would lead to a decrease in excitation, signaling or global firing activities. Accordingly, a lower activity has been reported in electrophysiological recordings of NCL neurons responding to familiar stimuli compared to novel stimuli in crows (Veit et al., 2015). In rabbits and rodents, it has been shown that the mPFC controls the performance of selected behaviors against unwanted ones by decreasing or increasing global firing activities of prefrontal projection neurons and that high frequency stimulation in the mPFC evokes significant long-term depression (Leal-Campanario et al., 2013; Gruart et al., 2015). Particularly, LTD is associated with both, the expression of the *GRIN2B* receptor subunit and the internalization of AMPA receptors (Brigman et al., 2013; Paoletti et al., 2013; Shin et al., 2020). Both receptors also play an important role in synaptic plasticity that is linked to associative learning (Gray et al., 2011; Paoletti et al., 2013; Mukherjee et al., 2017), which may explain decreased levels of *GRIN2B*, *GRIA2*, *GRIA3* and *GRIA4* in the NCL observed here. However, all glutamatergic receptors are also expressed in glia cells and proliferation and gliogenesis was increased in the medial prefrontal cortex of rats during learning of an operant stimulus-response task (Rapanelli et al., 2010). Because our tissue sampling method did not allow for differentiation between glia and neuronal cells, the glutamatergic receptor mRNA of glia cells might have additionally contributed to our measurements. So far, at least in mammals, it is known that astrocytes play an important role to regulate network plasticity and the function of neuronal networks (Mederos et al., 2018; Durkee and Araque, 2019; Lyon and Allen, 2021). In birds less is known about the role of glia cells during learning, but it was shown that a food caching bird like the blacked-capped chickadees exhibited more glial cells in the hippocampal formation if they had lived in the wild compared to lab reared birds (Roth et al., 2013). Roth and colleagues concluded that this may rely on individual experience and environment. Thus, the exact role of glia cell function in learning and memory in birds might be a very interesting topic for future research.

### Monoaminergic Receptors

The decrease in *DRD1A* and *DRD1D* receptors after SR training confirmed earlier results and is likely associated to regular bouts of rewards and dopaminergic stimulation in the NCL after extended periods of training (Herold et al., 2012). This assumption is further supported by expression analysis of D1 and D2 receptors in the nucleus accumbens of zebra finches that show relative expression changes with regard to the experience of a rewarding behavior (Alger et al., 2022).

The finding of a decrease of the noradrenergic receptor subtype *ADRA1A* after SR training adds a new aspect. In general, *ADRA1A* receptors are involved in certain aspects of synaptic plasticity, learning and memory like regulation of adult neurogenesis (which occurs in the nidopallium of birds (Melleu et al., 2016)), gliogenesis, LTP and LTD (Gupta et al., 2009; Perez, 2020). Apart from neurons, where *ADRA1* are expressed at all sides but predominantly at axon terminals (Mitrano et al., 2012), *ADRA1A* can be also expressed in stem cells or glia cells, while they were not detected *in vivo* in cerebral blood vessels, in contrast to the periphery where the receptor can be found in vascular smooth muscle (Papay et al., 2006; Perez, 2020). Thus, we cannot exclude that a small amount of our probe constitutes also stem cell and glia cell *ADRA1A* mRNA.

In birds, treatments with non-subtype-selective ADRA1 agonists have been shown to inhibit the consolidation of memory if applied to the intermediate medial mesopallium of chicks (Gibbs and Summers, 2001). In addition, application of low doses into the nucleus interfascialis of the nidopallium (NIf) of zebra finches increased the auditory response of neurons in the higher vocal center (HVC), which is a major target structure of Nlf neurons, while higher doses suppressed activity in both, NIf and HVC (Cardin and Schmidt, 2004). A non-subtype-selective ADRA1 antagonist infused in the NIf prevented the arousal mediated suppression of HVC auditory responses that demonstrated the critical involvement of these receptor types in attentional modulation (Cardin and Schmidt, 2004). Consistent with the latter finding it seems to be very likely that *ADRA1A* plasticity in the NCL is modulated by the attentional requirements of the SR task and that the decrease is a result of persistent training to pay attention to a certain stimulus. It may also modulate local as well as long range neural activity, but this has to be further studied as well as further noradrenergic receptor subtypes.

### Stimulus-Response Versus Simultaneous-Matching-To-Sample

In contrast to SR learning, adding new task components, i.e., training the discrimination between two stimuli, including processes like stimulus comparison and response selection (or decision-making), did not affect the expression profile of the analyzed receptors in the NCL differentially, when compared to the SR-group. Receptors in the SMTS-group were either decreased similar to the SR-training or showed no changes in expression levels like *GRIA2* and *DRD1A* as compared to the untrained pigeons. This is in line with earlier data for concentrations of different D1 receptor subtypes and the D2 receptor in the NCL and striatum (Herold et al., 2012). Therefore, we would assume that receptor plasticity in the NCL is more important for the initial operant training and consolidation of relevant reward-related stimulus information, but not to stimulus comparison and selection *per se* that may depend on other neural mechanisms. Finally, changes in favor of the SR-task compared to the SMTS-task, which has a higher cognitive load might also reflect a difference in encoding the reward value, which is guaranteed in the SR-task after pecking a visual stimulus, while the SMTS-task holds the chance to fail. This point would be in line with electrophysiological recordings that have shown that the encoding of the reward value was most prominent during the sample period, i.e., presentation of a visual key that is associated to reward (Dykes et al., 2018).

### Receptor Plasticity in the HF After Associative Learning and Stimulus Discrimination Training

The receptor subtype/subunit expression was also specifically altered after associative learning in the HF but revealed different expression dynamics compared to the NCL. In addition to the considerably plastic nature of the avian HF forming constantly new neurons in association with learning and memory and showing volume changes (Patel et al., 1997; Barnea and Pravosudov, 2011; Hall et al., 2014), the present study indicates that the function of the avian HF further relies on changes in neurotransmitter receptor expression. These findings confirm that receptor changes in the HF of birds are involved in training of an associative stimulus-reward task (Reilly and Good, 1989; Bingman, 1993). Control pigeons showed high mRNA levels of the glutamatergic NMDA receptor subunits *GRIN1* and *GRIN2B* and the AMPA receptor subunits *GRIA2* and *GRIA3*, while from the monoaminergic receptors, the dopaminergic *DRD1A* receptor subtype showed the highest concentration in the HF. This fits well with the receptor protein profile obtained by receptor autoradiography (Herold et al., 2014) and detection of all AMPA receptor types in the HF of pigeons (Rosinha et al., 2009). The low expression levels for the GABA_A_ receptor *GABARG2* subunit in the HF is also in line with a general lower receptor concentration of GABA_A_ receptors in the HF that was reported with Muscimol-binding to the *α*-subunits of the GABA_A_ receptor when compared to NMDA and AMPA receptors (Herold et al., 2014). The low mRNA levels of *5HTR1A* in the HF of pigeons support earlier findings of an *in-situ* hybridization study of expression patterns for *5HTR1A* in the brain of chicks (Fujita et al., 2020). The discrepancy between mRNA levels and protein levels of the HTR1A, which showed high densities of binding sites in both, HF and the NCL compared to other monoaminergic receptors, can be explained by the fact that the ligands access the protein levels binding both, pre- and postsynaptic sites, while *5HTR1A* mRNA cannot be detected in axon terminals (Vilaro et al., 2020). Overall, *GRIN1*, *GRIN2C*, *GRIA3*, *5HTR1A* and *DRD1A* levels were lower, *GRIN3B* and *GRIA1* were higher and *GRIN2B*, *GRIN3A*, *GABARG2*, *ADRA1A*, *DRD1B* (*DRD5*) and *DRD1D* were similarly expressed in the HF of the control group when compared to the NCL. These differences in receptor expression profiles are in line with the findings of individual receptor fingerprints for both brain areas (Herold et al., 2011; Herold et al., 2014).

### Glutamatergic NMDA2B Receptors

After training in the SR task, we found decreased levels for the glutamatergic subunit *GRIN2B* in the HF. Here, *GRIN2B* showed a comparable decrease to the NCL. Therefore, it could be argued that the decrease may serve a similar function for SR and SMTS in both regions. In mice, the loss of GRIN2B receptors in the hippocampus and cortex resulted in abolished NMDA-dependent LTD as well as in impairment in several cortico-hippocampal mediated tasks like T-Maze spontaneous alternation, water maze and pavlovian trace fear conditioning (Brigman et al., 2010; Shin et al., 2020). Additional findings in the hippocampus also described the critical contribution of GRIN2B for NMDAR channel function and the maintenance and formation of dendritic spines (Akashi et al., 2009). In humans, *GRIN2B* also plays a role in response inhibition which is mainly controlled by cortico-basal ganglia circuits (Beste et al., 2010). In our view, we can only speculate that the reduction in *GRIN2B* in the HF and the NCL could have facilitate general learning-dependent, but task-independent processes that were evoked by global glutamate release during the training that can result in decreased *GRIN2B* levels. This may be supported because in hippocampal slices of rats it was observed that specifically GRIN2B containing NMDA receptors monitor glutamate released from multiple sources, i.e., afferent inputs, and thus monitor the level of overall activity level in a network and regulate the strength of glutamatergic synapses (Scimemi et al., 2004). In contrast, *GRIN2A* containing NMDA receptors seem to be more important to sense “local” glutamate signaling (Scimeni et al., 2004) and so it would be of interest for future studies to investigate the expression of *GRIN2A* as well. In addition, electrophysiology studies revealed that although stimulus-responsive neurons have been detected in the HF of crows and pigeons (Scarf et al., 2016; Ditz et al., 2018), studies in the HF of pigeons have revealed less stimulus-specific neurons (Scarf et al., 2016). It has to be also considered that stimulus discrimination in the HF of pigeons is further highly associated with a spatial component (Coppola et al., 2014). Further, a new study suggested that the function of the HF in memorizing properties, or more precisely probabilities of reward occurrences, seems to be associated with encoding of the spatial location (Sizemore et al., 2021). Thus, this might also support the theory that a reduction of *GRIN2B* is more correlated to global behavioral learning phenomena.

### GABAergic GABA_A_ Receptors

It is well accepted that GABA_A_ receptor signaling is critically involved in regulating synaptic inhibition in the hippocampus of mammals and that the GABARG2 subunit is important for controlling efficacy of synaptic inhibition and spatial learning (Kittler et al., 2008; Tretter et al., 2009). Moreover, GABA_A_ receptors and expression of the *GABARG2* subunit is necessary for developmental neurogenesis as well as for adult neurogenesis in the hippocampus (Earnheart et al., 2007; Jacob et al., 2008). The present data of increased levels of *GABARG2* in the pigeons HF after SR as well as after SMTS training demonstrate that this might also apply for pigeons and perhaps more general, in birds. Whether the increase was a result of increased neurogenesis after learning or shaped the efficacy of synaptic inhibition needs to be further investigated. In fact, adult neurogenesis in the HF of birds was stimulated by learning and memory processes (Patel et al., 1997; Barnea and Pravosudov, 2011; Hall et al., 2014) as well as environmental enrichment (Melleu et al., 2016), which makes it likely that the training and stimulation of neurogenesis in the cognitive tasks played a role for increased *GABARG2* subunit expression in the pigeon HF. Because our probe also included glia cells that can express GABA_A_-receptors the increase might be partly induced by gliogenesis (Mederos and Perea, 2019). However, in rats it has been shown that learning and training of a rewarded operant conditioning task resulted only in increased astrogliogenesis in the medial prefrontal cortex, but not in the hippocampus where neurogenesis occurred (Rapanelli et al., 2011). The findings of Rapanelli and colleagues would then speak against a participation of glial *GRIN2B* subunit expression to the decrease observed in the HF here.

### Stimulus-Response Versus Simultaneous-Matching-To-Sample

Both, *GRIN2B* and *GABARG2* subunits were either reduced or increased after SMTS training comparable to the effects observed after SR training. Similar as for the NCL, no significant differences were observed between the SR and SMTS condition. This may imply that the behavioral training in general resulted in changes of receptor expression in the HF and NCL but changed the level of neurotransmitter receptors and their subunits differentially. According to our results the changes occurred mainly in favor of the SR-task. It has to be emphasized that the SMTS-task included all behavioral skills and cognitive requirements of the SR-task plus cognitive functions like stimulus comparison, response selection (i.e., decision-making) and reward evaluation (uncertainty). Therefore, it may be hypothesized that the absence of differences between SR and SMTS training suggests that neurotransmitter receptor changes in the NCL and the HF do not have such a high value for encoding functions like response selection or stimulus-comparison. Because damage to the pigeon hippocampus has been shown to have an impact on operant-conditioning (Good and MacPhail, 1994; Colombo et al., 2001), it is in line with our data that we found effects after the stimulus-response learning and after the SMTS-learning (because the operant-conditioning procedure preceded both tasks) but no effects between both SR- and SMTS. This would further correspond with lesion data that show that the hippocampus is not involved in visual-discrimination or response selection per se (see Colombo and Broadbent, 2000 for review and; Colombo et al., 2001). On the other hand, this might imply that synaptic processes that increase performance in those functions might be possibly driven by other neural mechanisms or factors like for example brain derived neurotrophic factor (BDNF) or cAMP response element binding protein (CREB) expression and do not underly plastic changes at the level of neurotransmitter receptors. Clearly, further research in the field is needed to elucidate this question.

## Conclusion

The data of the present study emphasize the role of NCL and HF in stimulus-response learning and related neurotransmitter receptor expression and plasticity in both brain structures. This adds new insights regarding information processing and learning mechanisms in the bird brain. Many parallels between mammalian and avian prefrontal and hippocampal structures have been elucidated for the first time including the expression of specific receptor subtypes and subunits as well as their role in stimulus-response learning and attentional processes in an avian model that is frequently used for comparative studies: the pigeon. The basic principles of learning seem to be conserved between species with some degrees of freedom may exist that besides adaption and specialization still result in incredible abilities of learning and memory of birds.

## Data Availability

The original contributions presented in the study are included in the article/Supplementary Material, further inquiries can be directed to the corresponding author.
